# Negative Impact of Amphetamine-Type Stimulant Use on Opioid Agonist Treatment Retention in Ontario, Canada

**DOI:** 10.3389/fpsyt.2021.782066

**Published:** 2021-12-20

**Authors:** Kristen A. Morin, Frank Vojtesek, Shreedhar Acharya, David C. Marsh

**Affiliations:** ^1^Marsh Research Lab, Northern Ontario School of Medicine, Sudbury, ON, Canada; ^2^ICES North, Sudbury, ON, Canada; ^3^Canadian Addiction Treatment Centre, Markam, ON, Canada; ^4^Health Sciences North Research Institute, Sudbury, ON, Canada

**Keywords:** Opioid Agonist Treatment, amphetamine-type stimulant use, rural health, treatment discontinuation, opioid use disorder

## Abstract

**Objective:** The objective of this study was to evaluate epidemiological trends of co-use patterns of amphetamine-type stimulants and opioids and the impact of co-use patterns on Opioid Agonist Treatment (OAT) retention in Ontario, Canada. The secondary objective was to assess geographical variation in amphetamine-type stimulant use in Northern Rural, Northern Urban, Southern Rural and Southern Urban Areas of Ontario.

**Methods:** A retrospective cohort study on 32,674 adults receiving OAT from ~70 clinics was conducted between January 1, 2014, and December 31, 2020, in Ontario, Canada. Patients were divided into four groups base on the proportion of positive urine drug screening results for amphetamine-type stimulants during treatment: group 1 (0–25%), group 2 (25–50%), group 3 (50–75%), and groups 4 (75–100%). A Fractional logistic regression model was used to evaluate differences over time in amphetamine-type stimulant use with urine drug screening results. A Cox Proportional Hazard Ratio model was used to calculate the impact of amphetamine-type stimulant use on retention in OAT and adjusted for sociodemographic characteristics, drug use and clinical factors. Lastly, a logistic regression model was used on a subgroup of patients to assess the impact of geography on amphetamine-type stimulant use in Northern Rural, Northern Urban, Southern Rural and Southern Urban Areas of Ontario.

**Results:** There were significant differences in amphetamine-type stimulant positive urine drug screening results year-over-year from 2015 to 2020. Significant differences were observed between amphetamine-type stimulant groups with regards to sociodemographic, clinical and drug use factors. Compared to those with no amphetamine-type stimulant use, the number of days retained in OAT treatment for amphetamine-type stimulant users was reduced (hazard ratio 1.19; 95% confidence interval = 1.07–1.17; *p* < 0.001). Lastly, an adjusted logistic regression model showed a significant increase in the likelihood of amphetamine-type stimulant use in Northern Rural regions compared to Southern Urban areas.

**Conclusion:** There was a significant increase in amphetamine-type stimulant use among individuals in OAT from 2014 to 2020, associated with decreased OAT retention. Research is required to determine if tailored strategies specific to individuals in OAT who use amphetamine-type stimulants can improve OAT outcomes.

## Introduction

Stimulant use disorder is the second most common illicit substance use disorder in the world after opioids (1). Recent studies from the United States have reported increased co-use patterns of stimulants and opioids in the past year (2, 3). In Canada, the estimated prevalence of stimulant use in the population is about 1%, with higher rates of use among youth (3.5%) and some of the highest rates in rural areas (4, 5). Polydrug use among individuals with opioid use disorder (OUD) has been shown to increase poisonings and fatal overdose rates (6–10).

Several studies have documented the efficiency of Opioid Agonist Treatment (OAT) to treat OUD, and its effectiveness increases the longer a patient is retained in treatment (11–13). Unfortunately, there are currently no effective pharmacological treatments for stimulant use disorders (14). Despite other modalities having shown efficiency for treating stimulant use disorder, such as contingency management and cognitive-behavioral therapies (CM/CBT) (15–17), such treatments are not routinely available for patients with OUD in Canada apart from contingency management approaches to take-home doses of OAT medication.

Acute Pharmacological effects of stimulant use are well-known to reduce impulse control (18). There is also literature demonstrating increased psychotic episodes, aggressive behavior and cognitive problems (19, 20) from long-term methamphetamine use. Considering the increase in stimulant use in North America (5, 21–23), we hypothesize that combined with opioid use; stimulants may contribute to the rising issues with patients being retained in OAT.

Despite the evidence of increased stimulant and opioid use patterns in the United States, to our knowledge, there are no studies examining the effects of stimulant use on OAT retention in Canada. At the time of publication, the literature in Canada focused primarily on prescription stimulant use or stimulant use in youth (5); the results of these studies lack information on stimulant use among individuals with OUD. With very little research into the use of stimulants and opioids, more specifically amphetamine-type stimulants, we don't have a clear understanding of its impact on OAT outcomes in Ontario and even less is known about geographical variations in such outcomes. The lack of such insight is a critical gap in the literature, as stimulant use has been rising in the general population (5). Therefore, this study aims to evaluate epidemiological trends of co-use patterns of amphetamine-type stimulants and opioids and assess the impact on OAT retention. The secondary objective was to measure how the geographical location of residents is impacting amphetamine-type stimulant use in Ontario, Canada.

## Methods

### Study Design and Setting

A retrospective cohort study was conducted based on electronic medical record (EMR) data from the largest organization providing OAT in Canada (~70 clinics) from January 1, 2014, to December 31, 2020. Standardized evidence-based best practice policies and operating procedures are in place within the clinic network, which limits the likelihood of treatment variability between sites. A total of 31,701 adults in OAT in Ontario, Canada, were included in the study. The study data was accessed remotely using a secure server. Patient identification was anonymized. The Laurentian University Research Ethics Board provided ethical approval for this study. The Strengthening the Reporting of Observational Studies in Epidemiology (STROBE) guidelines were used to write this manuscript (24).

### Study Population

OAT patients were followed from the first OAT dispensation or prescription to either the end of the study or loss to follow-up. All OAT recipients during the follow-up period were identified based on the presence of at least one OAT episode in the EMR. OAT exposure was defined as any receipt of methadone or buprenorphine/naloxone.

### Amphetamine-Type Stimulants Exposure Groups

The amphetamine-type stimulant exposure groups were created based on the proportion of positive urine drug screening (UDS) results for amphetamine-type stimulants. Patients were divided into the following four groups: group 1 (0–25%), group 2 (25–50%), group 3 (50–75%), and groups 4 (75–100%).

### Covariates

Patient's characteristics were measured at the time of the most recent OAT dispensation. Patient characteristics included age, sex, and geographic health care delivery region (North/South, RIO-2008 Index). Patient characteristics were chosen because they have been shown to impact OAT retention (25–27). The Ontario Medical Association (OMA) online Rurality Index of Ontario (RIO) score matching application program interface (API) was used to check RIO scores to postal codes. The health care at home API was used to corroborate Local Health Integration Network (LHIN) scores to postal codes (25). Patients with missing postal codes (*n* = 4,735) could not be included in the geographical analysis. Therefore, a subgroup analysis was conducted on a subset of the cohort (*n* = 27,939 patients). Patients were divided into four geographical regions for the subgroup analysis: Southern Urban, Southern Rural, Northern Urban, and Northern Rural. Northern regions were defined by LHIN 13 and 14. The North/South divide has been used in several peer review studies and reports (26, 27). Rural regions were defined as any region with a RIO score of 40 or higher (28).

Clinical factors were included as covariates to isolate the impact of stimulants on treatment retention. The measured clinical characteristics included: initial OAT medication (methadone or buprenorphine/naloxone), the total number of days retained in OAT, whether a patient's starting dose was above the median starting dose for the cohort (6 mg for buprenorphine/naloxone and 30 mg for methadone), if a patient's peak dose was above the peak dose for the cohort (14 mg for buprenorphine/naloxone and 70 mg for methadone), and urine drug screening (UDS) results for cocaine, fentanyl, cannabis, and all opioids other than fentanyl and the patient's OAT medication. UDS groups were created based on the proportion of positive UDS for each drug and divided into quadrants 0–25, 25–50, 50–75, and 75–100%. Urine drug screen results were obtained using The FaStep Assay (Trimedic Supply Network Ltd., Concord, Ontario, Canada) with results for assays detecting amphetamine or methamphetamine combined for amphetamine-type stimulant results and assays detecting morphine or oxycodone combined for other opioid results. Results for fentanyl, cannabis and cocaine are based on specific assays detecting fentanyl, THC and cocaine metabolites.

### Treatment Discontinuation

Treatment discontinuation was defined as an interruption in a continuous period of dispensed OAT medication lasting at least 5 days for methadone and at least 6 days for buprenorphine/naloxone (29).

### Statistical Analysis

The percentage of amphetamine-type stimulant positive UDS was calculated from 2014 to 2020 in Ontario. A Fractional logistic regression model was used to assess significant change year-over-year in amphetamine-type stimulant positive UDS across Ontario from 2014 to 2020.

A descriptive analysis was conducted to compare covariates, including patient characteristics, clinical and drug use factors between stimulant groups. Chi-square test was used for categorical variables and Wilcoxon Rank Sum test for continuous variables. All *p*-values <0.05 were considered significant.

A Cox Proportional Hazards model was run to determine the effect of amphetamine-type stimulant use on the treatment discontinuation. First, an unadjusted model was run. The model was then adjusted for the aforementioned covariates, including geography (*n* = 4,735 missing data points).

A subgroup analysis of patients with geographical variables was conducted on a subset of 27,939 patients who had complete geographical information available. A multinomial logistic regression model was used to assess the association between amphetamine-type stimulant use and geography in the subset of the cohort with geographical data available between four geographical regions (Northern Rural, Northern Urban, Southern Rural, and Southern Urban). The model was then adjusted for all the covariates, including patient characteristics, clinical and substance use factors. Statistical significance was reported with 95% confidence intervals.

## Results

Between January 1, 2013, and December 31, 2020, 31,701 patients were included in the study. Of these patients, 27,016 (85.22%) had 0–25% of their UDS positive for amphetamine-type stimulants, 1,322 (4.17%) had 26–50% of their UDS positive for amphetamine-type stimulants, 1,153 (3.64%) had 51–75% of their UDS positive for amphetamine-type stimulants, and 2,210 (6.97%) had 76–100% of their UDS positive for amphetamine-type stimulants. Chi-Squared test for heterogeneity and the Wilcoxon-Rank-Sum/Kruskal-Wallis test showed a significant difference in each covariate except sex (*p*-value = 0.50). The results are presented in Table 1.

**Table 1:** Patient characteristics, clinical factors and substance use behaviors, stratified by amphetamine-type use groups among 31,701 people in OAT in Ontario, Canada.

	**Positive urine drug screening (UDS) results for amphetamine-type stimulants**				
	**0–25%**	**25–50%**	**50–75%**	**75–100%**	***P*** **-value**
	***n*** **= 27,016** **(85.22%)**	***n*** **= 1,322** **(4.17%)**	***n*** **= 1,153** **(3.64%)**	***n*** **= 2,210** **(6.97%)**	
Sex n (%)					0.50
Male	16,570 (61.33%)	790 (59.76%)	723 (62.7%)	1,348 (61%)	
Female	10,448 (38.67%)	532 (40.32%)	430 (37.3%)	862 (39%)	
Mean age (STD)	36 (10.9)	35 (9.4)	35 (9.3)	35 (9.0)	0.02
Location of residence (4,769 missing)					<0.01
Southern Urban	16,692 (72.89%)	802 (72.64%)	707 (73.11%)	1,499 (76.48%)	
Southern Rural	899 (3.93%)	54 (4.89%)	46 (4.76%)	80 (4.08%)	
Northern Urban	4,183 (18.27%)	176 (15.94%)	147 (15.2%)	273 (13.93%)	
Northern Rural	1,127 (4.92%)	72 (6.52%)	67 (6.93%)	108 (5.51%)	
Mean days in study (standard deviation)	718 (833.7)	821 (798.7)	637 (782.9)	441 (687.4)	<0.01
Methadone starting medication n (%)	20,984 (77.67%)	1,068 (80.79%)	929 (80.57%)	1,760 (79.64%)	<0.01
Starting dose above median starting dose n (%)	12,889 (47.71%)	639 (48.34%)	514 (44.58%)	829 (37.51%)	<0.01
Peak dose above median peak dose n (%)	6,245 (23.12%)	343 (25.95%)	287 (24.89%)	419 (18.96%)	<0.01
Average monthly UDS group *n* (%)					
1 per month or less	718 (2.66%)	0 (0%)	1 (0.09%)	23 (1.04%)	<0.01
Bi-weekly per month	1,986 (7.35%)	11 (0.83%)	19 (1.65%)	246 (11.13%)	
Weekly	3,389 (12.54%)	69 (0.26%)	61 (5.29%)	166 (7.51%)	
More than weekly	20,923 (77.45%)	1,242 (93.95%)	1,072 (92.97%)	1,775 (80.32%)	
Cocaine UDS positive group *n* (%)					<0.01
0–25% positive	19,037 (70.47%)	2,451 (9.07%)	1,914 (7.06%)	3,614 (13.38%)	
25–50% positive	698 (52.8%)	232 (17.55%)	147 (11.12%)	245 (18.53%)	
50–75% positive	626 (54.29%)	181 (15.7%)	153 (13.27%)	193 (16.74%)	
75–100% positive	1,282 (58.01%)	344 (15.57%)	232 (10.5%)	352 (15.93%)	
Fentanyl UDS positive group *n* (%)					
0–25% positive	24,555 (90.89%)	801 (2.96%)	646 (2.39%)	1,014 (3.75%)	<0.01
25–50% positive	981 (74.21%)	108 (8.17%)	104 (7.87%)	129 (9.76%)	
50–75% positive	786 (68.17%)	86 (7.46%)	113 (9.8%)	168 (14.57%)	
75–100% positive	1,275 (57.69%)	170 (7.69%)	148 (6.7%)	617 (27.92%)	
Cannabis UDS positive group *n* (%)					
0–25% positive	17,444 (64.57%)	1,213 (4.94%)	1,230 (4.55%)	7,129 (26.39%)	<0.01
25–50% positive	603 (45.61%)	107 (8.09%)	100 (7.56%)	512 (38.73%)	
50–75% positive	582 (50.48%)	73 (6.33%)	96 (8.33%)	402 (34.82%)	
75–100% positive	1,382 (60%)	110 (4.98%)	105 (4.75%)	669 (30.27%)	
Other opioid UDS positive group *n* (%)					
0–25% positive	20,293 (75.11%)	2,920 (10.81%)	1,926 (7.13%)	1,877 (6.95%)	<0.01
25–50% positive	987 (74.66%)	214 (16.19%)	97 (7.34%)	24 (1.82%)	
50–75% positive	798 (69.12%)	210 (18.21%)	121 (10.49%)	24 (2.08%)	
75–100% positive	1,374 (62.17%)	403 (18.24%)	320 (14.48%)	113 (5.11%)	

**Figure 1:**
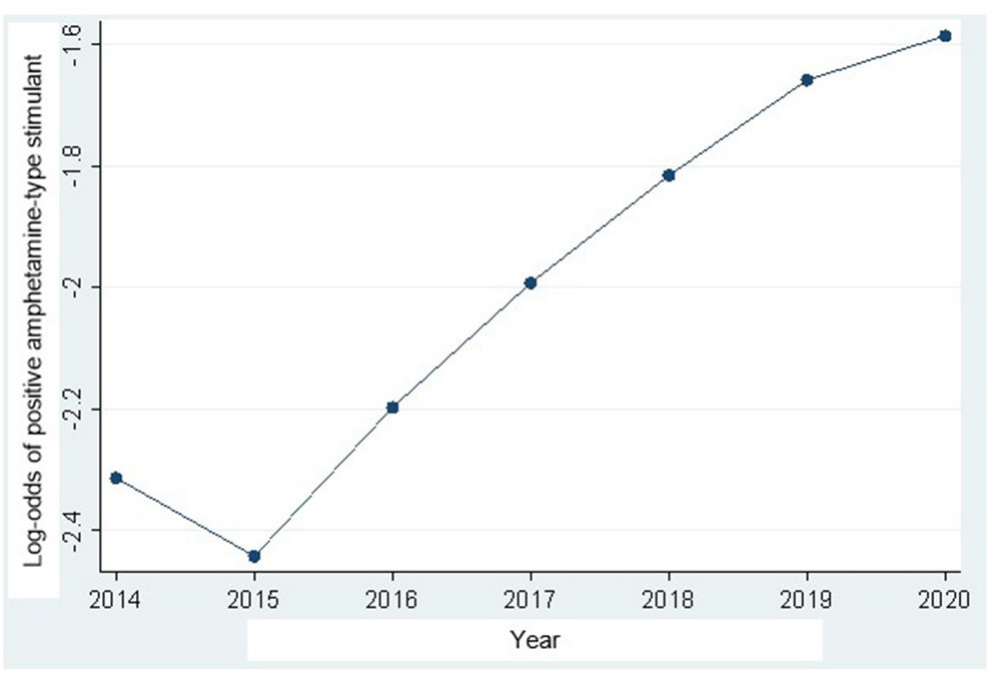
Amphetamine-type stimulant urine drug screening (UDS) results trajectory in Ontario Canada from 2014 to 2020 (detailed results available in Table 2).

In the trend analysis, the amphetamine-positive UDS results increased significantly during the study period 2014–2020. Interestingly, as shown in Figure 1, there was a decrease in amphetamine-positive UDS between 2014 and 2015, but after 2015, positive UDS results increased significantly until the end of the study period. Detailed results including 95% CI are available in Table 2.

**Table 2:** Odds ratios and 95%confidence intervals (95%CI) for amphetamine-type stimulant urine drug screening (UDS) in Ontario, Canada from 2014 (ref) to 2020.

**Year**	**Odds ratio**	**95% CI**
**2014 (ref)**		
**2015**	2.44	2.44–2.44
**2016**	2.2	2.19–2.20
**2017**	1.99	1.99–1.99
**2018**	1.82	1.81–1.81
**2019**	1.66	1.65–1.66
**2020**	1.59	1.58–1.59

### Outcome Results

**Figure 2:**
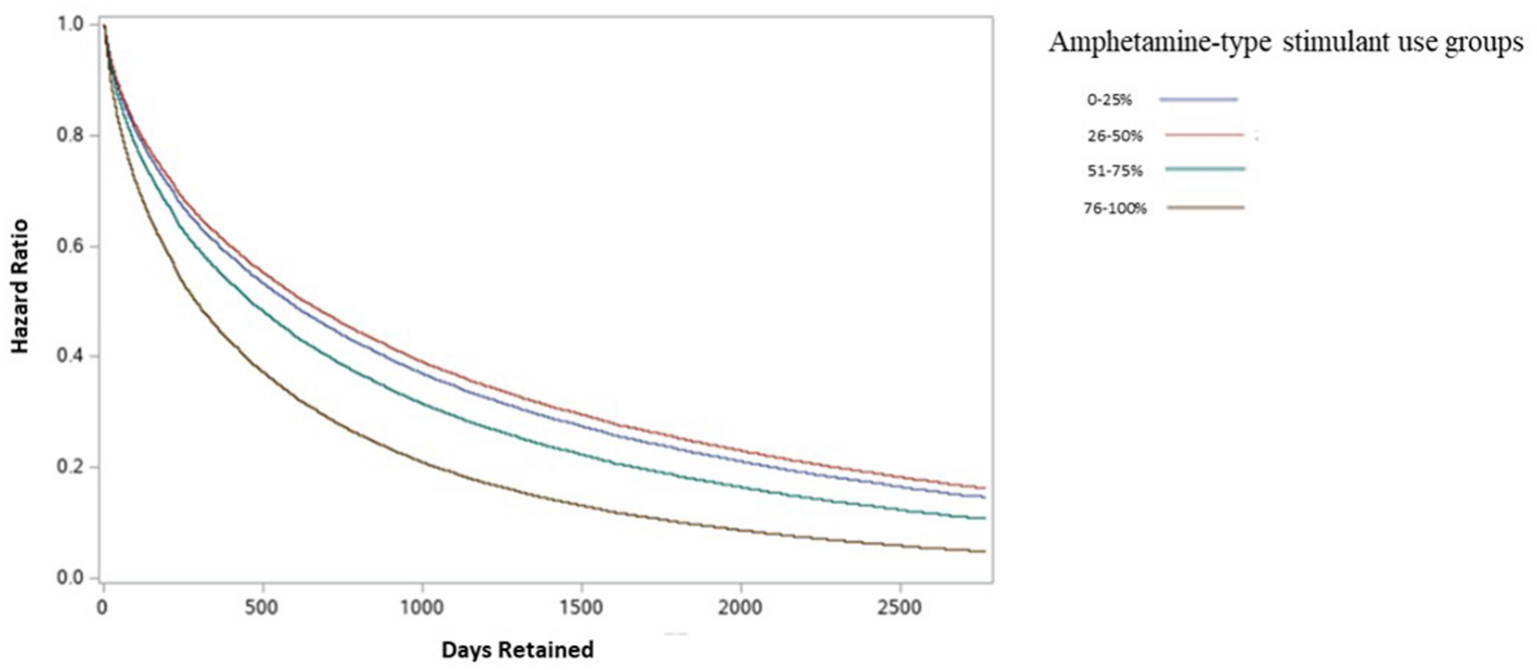
Adjusted discontinuation probability between amphetamine-type stimulant groups among individuals in OAT in Ontario, Canada.

The impact of amphetamine-type stimulant use on OAT discontinuation was assessed using a Cox proportional Hazard Model. Figure 2 shows the results of the adjusted Cox Proportional Hazard Ratio model. The model was adjusted for patient characteristics, clinical and drug use factors. The adjusted model showed no significant increase in treatment discontinuation rate in group 2 (patients with 26–50% positive amphetamine-type stimulant UDS) compared to group 1. However, there was a significant increase in treatment discontinuation rate in groups 3 (patients with 51–75% positive amphetamine-type stimulant UDS) (aHR = 1.160, 95% CI 1.078–1.248) and 4 (patients with 76–100% positive amphetamine-type stimulant UDS) (aHR = 1.570, 95% CI 1.489–1.655) when compared to group 1 (patients with 0–25% positive amphetamine-type stimulant UDS). Detailed results of adjusted and unadjusted HR are available in Table 3.

**Table 3:** Unadjusted and Adjusted discontinuation probability (Hazard Ratio) between the amphetamine-type stimulant group, patient characteristics, clinical and drug use factors among individuals in OAT in Ontario, Canada.

**Variable**	**Unadjusted** **hazard ratio**	**95% wald** **confidence interval**	**Adjusted** **hazard ratio**	**95% wald** **confidence interval**
Stimulants use (ref = 0–25%)				
25–50%	0.85	0.80–0.90	0.94	0.88–1.01
50–75%	1.10	1.03–1.17	1.16	1.08–1.25
75–100%	1.53	1.50–1.61	1.57	1.49–1.66
Sex (ref = Female)	1.06	1.03–1.09	1.15	1.12–1.19
Age	0.86	0.85–0.87	0.80	0.79–0.82
Geography (ref = Southern Urban)				
Southern Rural	0.84	0.79–0.90	1.01	0.94–1.088
Northern Urban	0.87	0.84–0.91	0.91	0.87–0.94
Northern Rural	0.80	0.75–0.85	0.81	0.76–0.87
Starting medication (ref = buprenorphine/naloxone)	0.70	0.68–0.72	0.71	0.68.73
Starting dose above median starting dose (ref = no)	0.61	0.60–0.63	0.75	0.72–0.77
Peak dose above median peak dose (ref = no)	0.57	0.55–0.59	0.81	0.77–0.84
Average UDS per month (ref = once per month or less)				
Bi-weekly	0.41	0.38–0.45	0.48	0.43–0.52
Weekly	0.08	0.08–0.090	0.11	0.10–0.13
More than weekly	0.09	0.08–0.09	0.10	0.09–0.10
Cocaine use (ref = 0–25%)				
25–50%	1.09	1.04–1.13	1.11	1.06–1.16
50–75%	1.47	1.40–1.54	1.35	1.29–1.42
75–100%	2.16	2.09–2.24	1.75	1.68–1.82
Fentanyl use (ref = 0–25%)				
25–50%	0.77	0.72–0.82	0.68	0.63–0.73
50–75%	1.12	1.04–1.21	0.91	0.84–0.99
75–100%	2.27	2.16–2.40	1.63	1.54–1.73
Cannabis use (ref = 0–25%)				
25–50%	0.39	0.36–0.41	0.42	0.39–0.45
50–75%	0.46	0.43–0.49	0.48	0.45–0.52
75–100%	0.46	0.45–0.48	0.51	0.49–0.52
Other opioid use (ref = 0–25%)				
25–50%	1.60	1.54–1.66	1.37	1.31–1.43
50–75%	2.60	2.49–2.72	2.00	1.91–2.10
75–100%	5.21	4.97–5.46	3.25	3.08–3.44

### Subgroup Analysis Results

The impact of geography on amphetamine-type stimulant use was evaluated on a subgroup of patients (*n* = 26,932) using the Southern Urban group as the reference group. Results are presented in Table 3. A total of 19,700 (73.15%) patients resided in a Southern urban region, 1,079 (4.01%) lived in a Southern rural area, 4,779 (17.74%) resided in a Northern urban area, 1,374 (5.10%) lived in a Northern rural region. After adjusting for patient characteristics, clinical and drug use factors, the results showed a significant association between living in Northern Rural areas and increased prevalence of amphetamine-type stimulant use compared to living in Southern Urban areas (aOR = 1.4, 95%CI 1.1–1.8 for patients with 26–50% positive amphetamine-type stimulant UDS; aOR = 1.6, 95%CI 1.2–2.1 for patients with 51–75% positive amphetamine-type stimulant UDS; aOR = 1.4, 95%CI 1.1–1.7 for patients with 76–100% positive amphetamine-type stimulant UDS). There was no significant difference in the prevalence of amphetamine-type stimulant use in Southern Rural or Northern Urban regions. The results are presented in Table 4.

**Table 4:** Subgroup analysis: unadjusted and adjusted multivariable logistic regression model of geographical location associated with amphetamine-type stimulant use groups among individuals in OAT in Ontario, Canada.

**Urine drug screening results for amphetamine-type stimulant groups**	^*^OR	**95% CI**	^*^aOR	**95% CI**
**Group 2: 25–50%**				
**Location of residence**				
Group 2: Southern Rural	1.3	0.9–1.7	1.2	0.9–1.6
Group 3: Northern Urban	0.9	0.7–1.0	0.8	0.7–0.9
Group 3: Northern Rural	1.3	1.0–1.7	1.4	1.1–1.8
Group 3: 50–75%				
**Location of residence**				
Group 2: Southern Rural	1.2	0.9–1.6	1.2	0.9–1.6
Group 3: Northern Urban	0.8	0.7–0.9	0.8	0.7–1.1
Group 3: Northern Rural	1.4	1.1–1.8	1.6	1.2–2.1
Group 4: 75–100%				
**Location of residence**				
Group 2: Southern Rural	1.0	0.8–1.3	1.0	0.8–1.3
Group 3: Northern Urban	0.7	0.6–0.8	0.8	0.7–0.9
Group 3: Northern Rural	1.1	0.9–1.3	1.4	1.1–1.7

*Geography reference group = Southern Urban*.

*Stimulant urine drug screening reference group = 0–25%*.

* *OR, Odds Ratio.*.

* *aOR, Adjusted Odds Ratio*.

## Discussion

This study sought to evaluate the epidemiological trends of co-use patterns of amphetamine-type stimulants and opioids and the impact on OAT retention in Ontario, Canada. Drawing on longitudinal data from the largest organization providing OAT in Canada, a distinct upward trajectory of amphetamine-type stimulant use among individuals in OAT was observed over 5 years. Individuals in OAT who used amphetamine-type stimulants displayed lower retention rates after adjusting for individual characteristics, drug use behaviors and clinical factors. Interestingly living in Northern Rural areas of Ontario was associated with an increased likelihood of amphetamine-type stimulant use.

There were significant differences between amphetamine-type stimulant groups for all patient characteristics, clinical and substance use factors except for sex. We observed that amphetamine-type stimulant use was more frequent in younger individuals. Amphetamine-type stimulant users in our study were more frequently started on methadone vs. buprenorphine/naloxone, and those who tested positive for other drugs, including cocaine, fentanyl, cannabis and other opioids. The findings in this study, including age, methadone patients and patients using other drugs, reflect the evidence that OAT has become more available to higher-risk individuals to reduce overdose deaths (30), particularly during the era of illicit fentanyl availability (31).

As shown in the trajectory plot in Figure 1, there was a gradually increasing frequency of amphetamine-type stimulant use between 2015 and 2020. This finding corresponds with international research showing increases in stimulant use over time (1, 22, 23). At the time of publication, the Canadian literature was limited and primarily focused on prescription stimulant use, which corresponds with our finding of increased use over time (5, 32). However, we were unable to quantify illicit vs. prescribed stimulant use in this study.

In the primary analysis, amphetamine-type stimulant use was found to be associated with higher treatment discontinuation rates. It is possible that these individuals had more exposure to behavioral and social stressors or that psychotic episodes, aggressive behavior and cognitive problems, which are more common among individuals who use amphetamine-type stimulants (19, 20), triggered early treatment discontinuation. Research has shown that treatment outcomes could be improved by incorporating integrated, comprehensive services such as behavioral therapy, psychosocial supports, mental health treatment and flexible models of care (33–35). Research is needed to explore whether such strategies are effective for individuals with a history of concurrent opioid and amphetamine-type stimulant use, particularly to improve retention in OAT.

In the secondary analysis, the geographical location of residence was observed to impact amphetamine-type stimulant use. Living in Northern Rural Ontario was associated with an increased likelihood of amphetamine-type stimulant use. This result is consistent with previous findings that people in OAT residing in rural areas have higher rates of cocaine use compared to urban areas (10). Earlier studies have concluded that OAT patients in the North were more likely to be retained in treatment (10, 36). The higher retention rates in the North seem counter-intuitive, given patients often have to travel long distances to access OAT-prescribing physicians and pharmacies (36). However, Eibl et al. (36) demonstrated that patients in the North were 41% less likely to terminate treatment prematurely than were Southern patients. Given that in this study, we found that Northern patients are more likely to use amphetamine-type stimulants and that stimulant use is associated with a higher risk of treatment discontinuation, more research is needed to understand the drivers of higher OAT retention in the North.

Some limitations require consideration. First, data entry and reporting errors are possibly associated with using EMR data for research. Second, although we considered various factors associated with treatment retention, there is potential for unmeasured confounding, including confounding related comorbidities (7, 8, 37), social and interpersonal factors (38–41) and clinical characteristics (42, 43) due to our study only having access to routinely collected data within the EMR. Use of opioids, cocaine, fentanyl, cannabis and amphetamine-type stimulants was detected solely on the results of immunoassay-based urine drug screening conducted for clinical care. It, therefore, might include false-positive or false-negative results. Confirmatory testing with more sensitive and specific laboratory techniques was not possible on the large volume of tests included within this study. Finally, some expert opinions have suggested that routine UDS testing, physician and structural characteristics reinforce a power dynamic and invite shame, stigma and judgment (44, 45). We were not able to account for such factors in our analysis.

## Conclusion

In summary, our study identified a significant upward trajectory of amphetamine-type stimulant use, which was more common in Rural Northern areas. The results demonstrated that there are apparent differences in OAT retention rates among individuals who use amphetamine-type stimulants. The findings of this study highlight the potential value of acquiring a better understanding of the impact of increased patterns of opioids and amphetamines and the associated impacts of such patterns on OAT outcomes. The methods and findings can be generalized to other areas with similar OAT policies and programs. Our results further suggest a need to develop more comprehensive treatment strategies specific to people with different drug use patterns and geographical locations to maximize the benefits of OAT.

## Data Availability Statement

The datasets presented in this article are not readily available because the datasets contain identifiable confidential patient information and cannot be shared with anyone approved by the Research and Ethics Board. Requests to access the datasets should be directed to Kristen Morin kmorin@nosm.ca.

## Ethics Statement

The studies involving human participants were reviewed and approved by Laurentian University Research and Ethics Board. Written informed consent for participation was not required for this study in accordance with the national legislation and the institutional requirements.

## Author Contributions

KM: conceptualization, methodology, investigation, analysis, writing—original and final draft, and submission. FV: methodology, investigation, analysis, and writing. SA: investigation, methodology, and writing. DM: investigation, writing—review and editing, and supervision. All authors contributed to the article and approved the submitted version.

## Author Disclaimer

The analyses, conclusions, opinions, and statements expressed herein are solely those of the authors and do not reflect the funding or data sources; no endorsement is intended or should be inferred.

## Conflict of Interest

The authors declare that the research was conducted in the absence of any commercial or financial relationships that could be construed as a potential conflict of interest.

## Publisher's Note

All claims expressed in this article are solely those of the authors and do not necessarily represent those of their affiliated organizations, or those of the publisher, the editors and the reviewers. Any product that may be evaluated in this article, or claim that may be made by its manufacturer, is not guaranteed or endorsed by the publisher.
